# Large herbivores facilitate the persistence of rare taxa under tundra warming

**DOI:** 10.1038/s41598-022-05388-4

**Published:** 2022-01-25

**Authors:** Eric Post, Christian Pedersen, David A. Watts

**Affiliations:** 1 Department of Wildlife, Fish, and Conservation Biology, University of California Davis, One Shields Avenue, Davis, CA 95616 USA; 2 Department of Landscape Monitoring, Norwegian Institute of Bioeconomy Research, 1431 Ås, Norway; 3 Alaska Department of Health and Social Services, Division of Public Health, Alaska State Public Health Virology Laboratory, Fairbanks, AK 99775 USA

**Keywords:** Ecology, Biodiversity, Climate-change ecology, Community ecology

## Abstract

Ecological rarity, characterized by low abundance or limited distribution, is typical of most species, yet our understanding of what factors contribute to the persistence of rare species remains limited. Consequently, little is also known about whether rare species might respond differently than common species to direct (e.g., abiotic) and indirect (e.g., biotic) effects of climate change. We investigated the effects of warming and exclusion of large herbivores on 14 tundra taxa, three of which were common and 11 of which were rare, at an inland, low-arctic study site near Kangerlussuaq, Greenland. Across all taxa, pooled commonness was reduced by experimental warming, and more strongly under herbivore exclusion than under herbivory. However, taxon-specific analyses revealed that although warming elicited variable effects on commonness, herbivore exclusion disproportionately reduced the commonness of rare taxa. Over the 15-year duration of the experiment, we also observed trends in commonness and rarity under all treatments through time. Sitewide commonness increased for two common taxa, the deciduous shrubs *Betula nana* and *Salix glauca*, and declined in six other taxa, all of which were rare. Rates of increase or decline in commonness (i.e., temporal trends over the duration of the experiment) were strongly related to baseline commonness of taxa early in the experiment under all treatments except warming with grazing. Hence, commonness itself may be a strong predictor of species’ responses to climate change in the arctic tundra biome, but large herbivores may mediate such responses in rare taxa, perhaps facilitating their persistence.

## Introduction

While rarity is a common state in nature, its converse, commonness, is unusual^1,2^. This dichotomy of states is evident across scales of organization, with most biomes, ecosystems, and local communities comprising a few common species and many rare ones^2,3^. For instance, on a global scale, as many as 37% of vascular plant species can be classified as very rare^3^. While rare species tend to display a combination of predictable attributes, including habitat specialization, small local population size, and limited geographic ranges^4^, understanding how rare species persist despite being rare has remained a challenge in ecology for decades^5,6^.

Alternatively, threats to rare species are well identified. Human land use and direct exploitation are commonly identified as primary threats to biodiversity via direct and indirect adverse effects on rare or geographically constrained taxa^7,8^. Additionally, however, numerous recent syntheses and meta-analyses have emphasized that rare species, or species with some aspect of rarity such as low abundance or restricted distributions, are at greatest risk of extinction due to effects of climate change on local bioclimatic suitability or habitat availability^9–12^. Conversely, climatic stability may facilitate regional diversity through maintenance of greater numbers of rare species than are found in climatically unstable regions^3^.

Species interactions may also be important in maintaining rare species in local assemblages. Common taxa, for instance, are typically exploited by a greater abundance and diversity of natural enemies such as consumers and pathogens^2^. And exploitation of common species can be important in prevention of competitive exclusion of rarer or less abundant species^13^. In arctic grazing systems, for instance, removal of a keystone herbivore, especially in combination with warming, can rapidly erode local diversity as competitively dominant plant species increase in abundance or occurrence and rare species are lost from the local assemblage^14,15^. An anticipated consequence of climate change and altered species interactions in such systems, therefore, may be that common species will become more common while rare species become increasingly rare.

In comparison to temperate and tropical systems, species diversity is characteristically low in the Arctic^16^, where warming is also occurring at a rate 2–3 × the global average^17,18^. Warming-driven invasion of tundra by woody plant species^19,20^, and increases in abundance or occurrence of already common tall statured tundra species^21^, may reduce abundances of less competitive, smaller statured species^14,21^. Browsing, trampling, and fecal and urinary nitrogen inputs by large herbivores can interact in important ways with warming-associated shifts in tundra plant community composition and diversity^14,22^. Abundance responses to alteration of consumer pressure or abiotic conditions do not by necessity, however, result in changes in commonness or rarity, which are relative. Hence, inferences about implications for changes in commonness that are drawn from studies focusing on absolute abundance responses to climate change or altered exploitation may be of limited value. Consequences for commonness and rarity of arctic tundra taxa of interactive effects of warming and herbivory therefore warrant explicit investigation. Here, we report results from a 15-year warming and herbivore exclusion experiment at an arctic site, the last 12 years of which focused on their interactive effects on commonness and rarity of 14 tundra taxa within the community. Previous experimental work at an arctic site in Finnish Lapland revealed that herbivore exclusion and warming nearly doubled the probability of loss of the rarest plant taxa from local assemblages, and that this loss probability attenuated with increasing commonness^14^. Accordingly, we here test the hypothesis that common and rare tundra taxa may respond in opposing fashion to warming and herbivore exclusion, with the related prediction that grazing by large herbivores may counteract adverse responses of rare taxa to warming. Insights from this study should thereby provide novel insights into factors contributing to the persistence of rare taxa in a biome undergoing rapid climate change.

## Results

### Commonness and rarity of the focal taxa at the study site

Across the 50 experimental plots, sitewide commonness of the 14 focal taxa was strongly right-skewed in the baseline year of assessment, 2006 (skewness = 2.25 ± 0.60; 95% confidence interval = 1.05, 3.45) and for the entire period of assessment, 2006–2017 (skewness = 2.16 ± 0.19; 95% confidence interval = 1.78, 2.54). Accordingly, 11 taxa (78.5% of the total) were classified as rare, with seven of these (50% of the total) classifiable as very rare, while only 3 taxa were classified as common across the study site (Table 1). All forbs, bryophytes, lichens, and fungi at the site were rare, while deciduous shrubs and graminoids were common (Table 1).

**Table 1:** Classification of tundra taxa at the study site near Kangerlussuaq, Greenland as rare or common according to descriptive statistics calculated across 50 experimental plots annually for the period 2006–17.

Taxon	Functional group	Classification	Mean (± 1SE) commonness	Minimum commonness	Maximum commonness
*Betula nana*	Deciduous shrub	Common	0.348 ± 0.01	0.296	0.401
Graminoids	Grass, rush, sedge	Common	0.245 ± 0.03	0.099	0.357
*Salix glauca*	Deciduous shrub	Common	0.095 ± 0.007	0.066	0.135
*Equisetum arvense*	Forb	Rare	0.013 ± 0.003	0.004	0.042
*Aulacomnium* sp.	Bryophyte	Rare	0.007 ± 0.001	0.004	0.013
*Stellaria longipes*	Forb	Rare	0.002 ± 0.008	4.0 × 10^–4^	8.44 × 10^–3^
*Cerastium alpinum*	Forb	Rare	0.001 ± 0.0004	0.0002	0.004
*Bistorta vivipara*	Forb	Very rare	4.17 × 10^–4^ ± 1.29 × 10^–4^	1.32 × 10^–5^	1.29 × 10^–3^
*Draba nivalis*	Forb	Very rare	2.05 × 10^–4^ ± 5.5 × 10^–5^	0	6.25 × 10^–4^
*Campanula gieseckiana*	Forb	Very rare	1.92 × 10^–4^ ± 7.58 × 10^–5^	0	9.13 × 10^–4^
*Viola canina*	Forb	Very rare	1.66 × 10^–4^ ± 6.73 × 10^–5^	0	6.81 × 10^–4^
*Peltigera* sp.	Lichen	Very rare	6.56 × 10^–5^ ± 4.77 × 10^–5^	0	5.83 × 10^–4^
*Pyrola grandiflora*	Forb	Very rare	2.54 × 10^–6^ ± 1.36 × 10^–6^	0	1.53 × 10^–5^
*Calvatia cretacea*	Fungus	Very rare	1.66 × 10^–6^ ± 1.22 × 10^–6^	0	1.32 × 10^–5^

### Functional group responses to 15 years of experimental warming and herbivore exclusion

**Figure 1:**
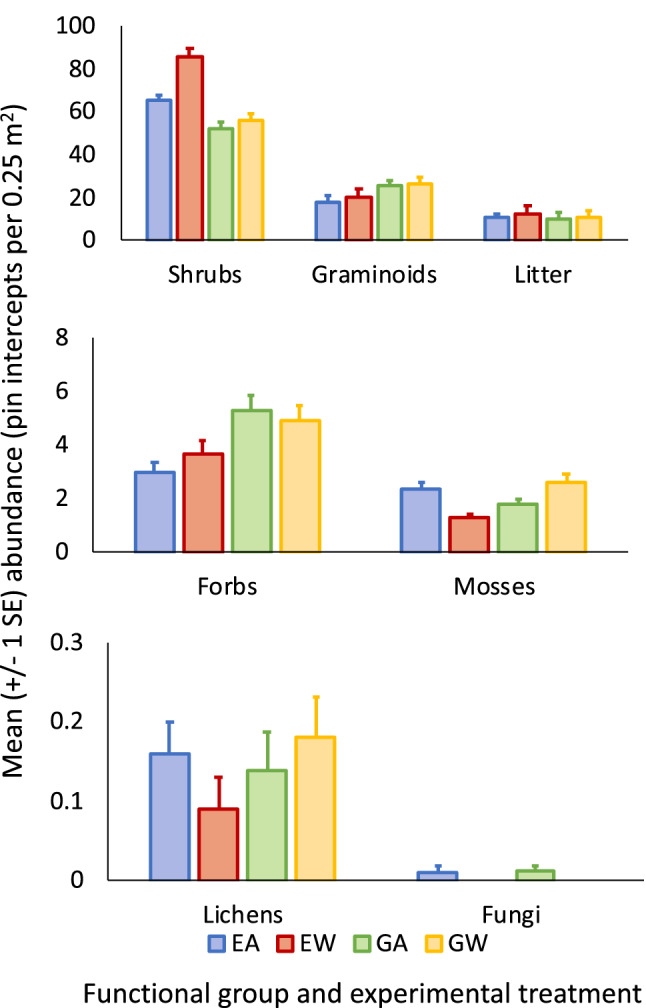
Mean (± 1 SE) abundance of arctic tundra functional groups and leaf litter at the study site near Kangerlussuaq, Greenland, by experimental treatment (2003–2017). EA = exclosed ambient, EW = exclosed warmed, GA = grazed ambient, and GW = grazed warmed.

Experimental warming reduced the abundance of the rarest functional group at the study site, fungi (Wald Chi-square = 4.14, *P* = 0.04; Fig. 1). Conversely, warming increased the abundance of the most common functional group at the study site, deciduous shrubs (Wald Chi-square = 25.0, *P* < 0.001; Fig. 1). Warming did not, however, alter the abundance of the second most abundant functional group, graminoids (Wald Chi-square = 0.77, *P* = 0.38; Fig. 1). Nor did warming alter the abundance of the other rare functional groups, forbs (Wald Chi-square = 0.1, *P* = 0.76), mosses (Wald Chi-square = 0.44, *P* = 0.51), or lichens (Wald Chi-square = 0.09, *P* = 0.76) (Fig. 1). As noted in the Methods, the lone fungus species, *C. cretacea*, did not occur on warmed plots, but did appear on ambient plots several years after the initiation of the experiment; hence, any inference about the effect of the warming treatment on the abundance (or commonness, as reported in the next sub-section) of this taxon must be drawn with caution.

Herbivore exclusion itself significantly reduced the abundance of one rare functional group, forbs (Wald Chi-square = 13.8, *P* < 0.001), marginally reduced the abundance of another rare functional group, mosses (Wald Chi-square = 3.47, *P* = 0.06), but did not significantly alter the abundance of two other rare functional groups, lichens (Wald Chi-square = 0.75, *P* = 0.39) and fungi (Wald Chi-square = 0.009, *P* = 0.92) (Fig. 1). Herbivore exclusion increased the abundance of one common functional group deciduous shrubs (Wald Chi-square = 79.6, *P* < 0.001), but reduced the abundance of another common functional group, graminoids (Wald Chi-square = 25.4, *P* < 0.001) (Fig. 1). Litter abundance increased in response to both warming (Wald Chi-square = 7.53, *P* = 0.006) and herbivore exclusion (Wald Chi-square = 13.4, *P* < 0.001) (Fig. 1).

Warming and herbivore exclusion interacted in altering shrub abundance (Wald Chi-square = 12.1, *P* < 0.001), with a greater increase in shrub abundance under warming and herbivore exclusion than under warming and grazing (Fig. 1). Likewise, moss abundance also responded to the interaction between warming and herbivore exclusion (Wald Chi-square = 20.8, *P* < 0.001), with warming reducing moss abundance under herbivore exclusion but increasing moss abundance under grazing (Fig. 1). There was no apparent interaction between the two treatments for abundance of graminoids (Wald Chi-square = 0.29, *P* = 0.59), forbs (Wald Chi-square = 1.22, *P* = 0.27), lichens (Wald Chi-square = 1.59, *P* = 0.21), fungi (Wald Chi-square = 0.007, *P* = 0.93), or litter (Wald Chi-square = 3.02, *P* = 0.08) (Fig. 1). However, the lowest mean abundance of two rare functional groups, mosses and lichens, both occurred under warming with herbivore exclusion (Fig. 1). Conversely, the only instance in which the greatest mean abundance of a functional group occurred under warming and herbivore exclusion was for the most common functional group, shrubs (Fig. 1).

### Alteration of commonness and rarity by experimental warming and herbivore exclusion

The GLM of commonness data pooled for all taxa, and that included taxon as a factor, revealed a marked overall reduction of commonness across the community by experimental warming (Wald Chi-square = 11.3, *P* = 0.001). Although herbivore exclusion did not alter pooled commonness across the community (Wald Chi-square = 0.53, *P* = 0.47), it interacted significantly with warming (Wald Chi-square = 5.81, *P* = 0.02). Consequently, warming reduced pooled commonness more strongly under herbivore exclusion than under grazing (exclosed ambient mean = 0.060 ± 0.002 vs. exclosed warmed mean = 0.047 ± 0.002; grazed ambient mean = 0.053 ± 0.002 vs. grazed warmed mean = 0.051 ± 0.002). An interaction among the warming treatment, herbivore exclusion treatment, and taxon (Wald Chi-square = 1367.8, *P* < 0.001) indicated that taxa responded individualistically to the experiment.

**Figure 2:**
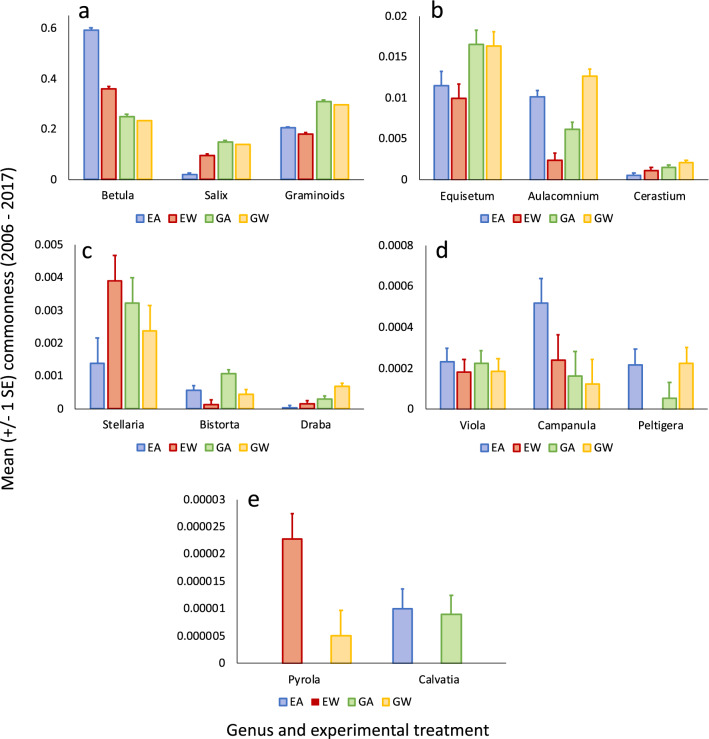
Mean (± 1SE) commonness by experimental treatment for each of 14 arctic tundra taxa at the study site near Kangerlussuaq, Greenland for the period 2006–2017, estimated from a generalized linear model with experimental treatment and year as factors.

Accordingly, taxon-specific GLMs of commonness revealed that experimental warming significantly reduced commonness of four taxa, two of them common (the deciduous shrub *Betula nana* and graminoids) and two of them rare (the forb *B. vivipara* and the fungus *C. cretacea*); and significantly increased commonness of four additional taxa, one of them common (the deciduous shrub *Salix glauca*) and three of them rare (the forbs *C. alpinum*, *D. nivalis*, and *P. grandiflorum*) (Fig. 2; Wald Chi-square statistics are provided in the Supplemental Table S1).

Experimental herbivore exclusion significantly reduced commonness of seven taxa, two of them common (the deciduous shrub *S. glauca* and graminoids) and five of them rare (the forbs *E. arvense, C. alpinum, B. vivipara, and D. nivalis*; and the moss *Aulacomnium* sp.), and increased commonness of two taxa, one of them common (the deciduous shrub *B. nana*) and one of them rare (the forb *C. gieseckiana*) (Fig. 2, and Supplemental Table S1). Hence, the commonness of rare taxa was disproportionately reduced by herbivore exclusion compared to common taxa.

The interaction between warming and herbivore exclusion influenced commonness of five taxa: *B. nana*, *S. glauca*, *Aulacomnium* sp., *S. longipes*, and *Peltigera* sp. (Supplemental Table S1). Warming reduced commonness of *B. nana* under herbivore exclusion but not under grazing; warming increased commonness of *S. glauca* under herbivore exclusion but not under grazing; warming reduced commonness of the moss *Aulacomnium* sp. under herbivore exclusion but increased its commonness under grazing; warming increased commonness of the forb *S. longipes* under herbivore exclusion and reduced it under grazing; and warming reduced commonness of the lichen *Peltigera* sp. under herbivore exclusion but increased its commonness under grazing (Fig. 2).

### Trends in commonness and skewness

**Figure 3:**
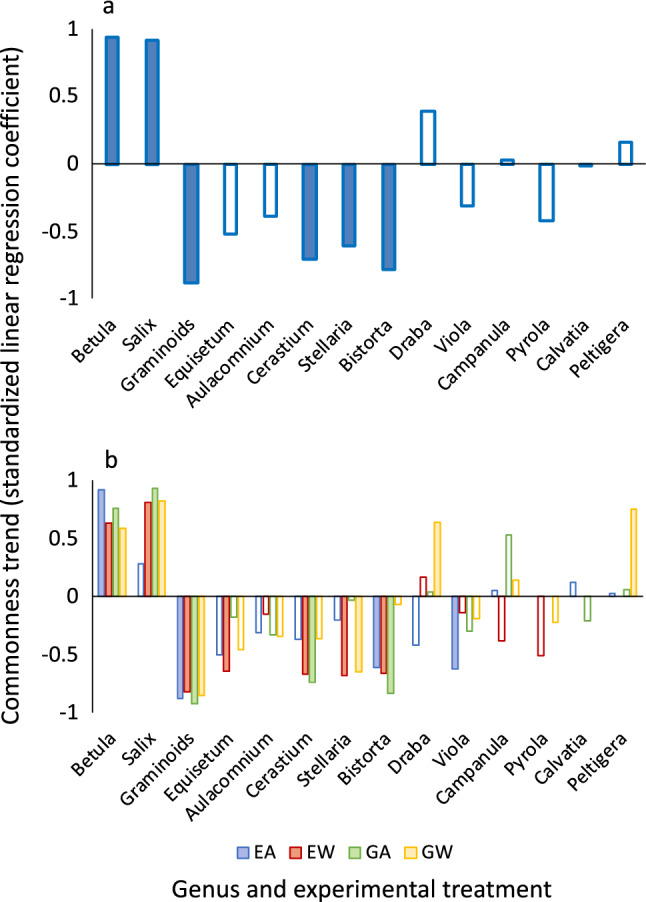
Trends in commonness of 14 arctic tundra taxa over the duration of a 12-year warming and herbivore exclusion experiment near Kangerlussuaq, Greenland, from 2006 to 2017. Panel (**a**) shows standardized linear regression coefficients for the regression of sitewide commonness vs. year for each taxon across all 50 plots at the study site. Panel (**b**) shows standardized linear regression coefficients for the regression of treatment-specific commonness vs. year for each taxon across all plots within each experimental treatment combination (EA = exclosed ambient, EW = exclosed warmed, GA = grazed ambient, and GW = grazed warmed). Solid columns indicate significant trends (*P* ≤ 0.05).

Over the 12-year period throughout which taxon-specific assessments were conducted, sitewide commonness of 2 common taxa (*B. nana* and *S. glauca*) increased, and sitewide commonness of one common and three rare taxa (graminoids, *C. alpinum*, *Stellaria longipes*, *B. vivipara*) declined, while sitewide commonness of the remaining eight taxa did not undergo clear trends in either direction (Fig. 3a). Thus, the only taxa to increase in commonness across the site over the course of the experiment were both common (the two deciduous shrubs), while declines occurred in graminoids and forbs.

Examination of trends within experimental treatments revealed increases in commonness over the 12-year period by the two deciduous shrubs, *B. nana* and *S. glauca*, under all treatment combinations except for *S. glauca* under the exclosed ambient treatment, where there was no apparent trend (Fig. 3b). Commonness increased through time for only two other taxa, the forb species *D. nivalis* and the lichen genus *Peltigera* sp., both under the grazed warmed treatment (Fig. 3b). In six of the remaining ten taxa, commonness declined through time under at least one treatment combination, without increasing through time for any of them (Fig. 3b). In five of these taxa (graminoids; the forbs *E. arvense*, *C. alpinum*, and *B. vivipara*; and the moss *Aulacomnium* sp.), commonness declined over time under the exclosed warmed treatment (Fig. 3b). Hence, warming under herbivore exclusion elicited more trends in commonness over the course of the experiment than any other treatment combination, with positive trends only for two common taxa (both deciduous shrubs) and negative trends for five taxa, including graminoids, which were common, and forbs and mosses, which were rare.

**Figure 4:**
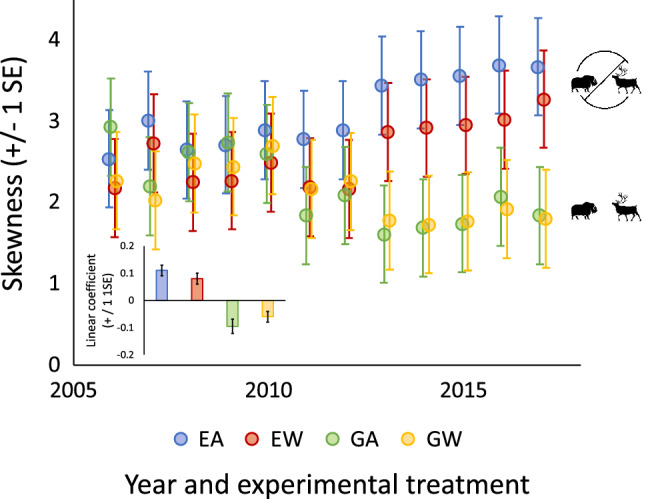
Time series of annual skewness (± 1SE) of the distribution of commonness of 14 arctic tundra taxa at the study site near Kangerlussuaq, Greenland from 2006 to 2017 by experimental treatment. Linear model coefficients (the slope of skewness vs. year) for each treatment are shown in the inset. EA = exclosed ambient (*R*^2^ = 0.81, *b* = 0.11 ± 0.02, *P* < 0.001), EW = exclosed warmed (*R*^2^ = 0.58, *b* = 0.08 ± 0.02, *P* = 0.004), GA = grazed ambient (*R*^2^ = 0.58, *b* = − 0.10 ± 0.03, *P* = 0.004), and GW = grazed warmed (*R*^2^ = 0.46, *b* = − 0.06 ± 0.02, *P* = 0.02).

As a consequence of differential responses of common and rare taxa to the experimental treatment combinations, distributions of commonness became increasingly right-skewed under herbivore exclosure and less right-skewed under herbivory (Fig. 4). A comparison of trends across treatment combinations revealed that skewness increased most under exclosed ambient conditions and declined most under grazed ambient conditions (Fig. 4 inset). By the end of the experiment in 2017, skewness was greatest on exclosed ambient plots, and lowest on grazed warmed and grazed ambient plots, and these latter two did not differ (Fig. 4).

**Figure 5:**
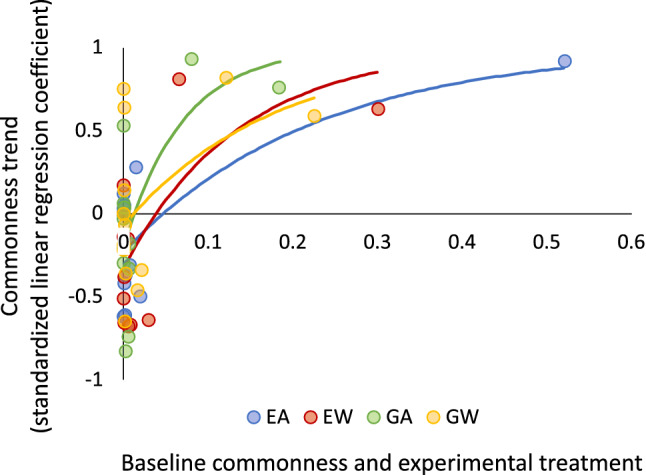
Nonlinear associations between trends in commonness of 13 arctic tundra taxa at the study site near Kangerlussuaq, Greenland, over the course of a 12-year warming and herbivore exclusion experiment (2006–2017) and their baseline commonness at the site in 2006. Lines are von Bertalanffy curves fit to the data using coefficient estimates from each treatment-specific model. EA = exclosed ambient (*R*^2^ = 0.66, *F*_2,11_ = 7.39, *P* = 0.009), EW = exclosed warmed (*R*^2^ = 0.45, *F*_2,11_ = 4.67, *P* = 0.03), GA = grazed ambient (*R*^2^ = 0.50, *F*_2,11_ = 4.74, *P* = 0.03), and GW = grazed warmed (*R*^2^ = 0.39, *F*_2,11_ = 1.88, *P* = 0.20).

With the exclusion of graminoids, a taxon that comprised at least eight genera within the experimental plots (see “Methods” section), trends in taxon-specific commonness were positively and non-linearly related to their baseline commonness in 2006 in all treatment combinations except the grazed warmed treatment (*F*_2, 11_ = 1.88, *P* = 0.20; Fig. 5). With the exception of the lichen *Peltigera* sp. under the grazed warmed treatment, all of the rare taxa declined under at least one treatment combination over the course of the experiment (Fig. 3), and their rates of decline scaled with their baseline commonness, i.e., rarer taxa tended to show steeper declines in commonness (Fig. 5). In contrast, the two common taxa, both deciduous shrubs, increased in commonness over the experimental duration under all treatment combinations, except for *S. glauca* under the exclosed ambient treatment (Fig. 3), and their rate of increase scaled with their baseline commonness under all treatment combinations. Hence, baseline commonness or rarity appeared to be a consistent predictor of increases in commonness and rarity over the last 12 years of the experiment, except under warming with grazing. Under this treatment combination, the pattern of trends toward increases in commonness of common taxa and declines in commonness of rare taxa evident under the other treatments was confounded by increases in commonness of two rare taxa, the forb *D. nivalis* and the lichen *Peltigera* sp. (Figs. 3, 5).

## Discussion

Local and site-specific patterns of commonness and rarity are not reliable indicators of commonness or rarity at distributional scales because most species are common locally but rare globally^23^. With that important caveat in mind, our results provide valuable insights into potential implications of ongoing and future climatic warming, changes in populations and communities of large herbivores, and their interaction, for commonness and rarity of arctic tundra taxa. The arctic tundra biome is relatively low in plant diversity, and our data indicate that nearly eighty percent of the taxa in this study can be classified as rare on our experimental plots. Among the seven taxa classifiable as very rare in this study, five (i.e., 36%) are vascular plants (Table 1). Hence, the proportion of vascular plant taxa discussed here that are very rare is comparable to that on a global scale^3^. A considerable unknown as the Arctic warms is whether tundra plant diversity will increase, presumably as a consequence of invasion by lower-latitude taxa^24^, or decrease, presumably as a consequence of increasing dominance by extant large-statured, resource-acquisitive taxa^14,25^. Nonetheless, warming-driven losses may ultimately outpace warming-driven gains in local plant diversity, especially in moisture-limited regions^26^ such as the Kangerlussuaq study site^27^. Indeed, at the Kangerlussuaq site, constraints on colonization by lower-latitude taxa imposed by multiple biogeographic barriers (including geographic isolation of the island of Greenland itself, as well as local mountains like Tasersiap Sermia and the Maniitsoq ice cap) enhance the potential for net reductions in plant diversity at the scale of our experimental plots. Moreover, the role of large herbivores in such dynamics is also difficult to predict, but some experimental and long-term observational evidence suggests grazing may maintain tundra plant diversity by preventing exclusion of less common taxa by tall and broad-canopied shrubs^14,15,28,29^.

We found mixed support for our hypothesis. Warming reduced pooled commonness across taxa, and it did so more strongly under herbivore exclusion than under grazing, but responses to warming by individual taxa were not consistently related to commonness or rarity. Conversely, responses to herbivore exclusion provided the most consistent support for our hypothesis. Herbivore exclusion reduced the commonness of half of the focal taxa, and five of these (71%) were rare. Hence, rare taxa were most consistently adversely affected by herbivore exclusion. Furthermore, warming in the absence of large herbivores reduced the commonness of two rare taxa while increasing the commonness of only one rare taxon. Moreover, both of the rare taxa for which commonness was reduced by warming under herbivore exclusion also increased in commonness under warming with grazing.

The abundance responses of functional groups across the entire 15 years of our experiment mirror those of many other warming and/or herbivore exclusion experiments and observational studies, including increases in shrub abundance and declines in graminoids, forbs, and nonvascular plants in response to warming, herbivore exclusion, or their combination^14,28,30–33^. However, abundance responses to experimental or observed variation in abiotic or biotic environmental conditions are not inherently indicative of commonness, which integrates both abundance and occurrence^3,34^. With few exceptions^14^, previous investigations of tundra plant responses to observed or experimental changes in climate or herbivory have focused less on aspects of commonness, and often consider commonness or rarity only qualitatively^30,35^.

In this context the following results of our experiment are particularly notable. First, warming exerted less consistent effects on commonness than did herbivore exclusion, which reduced the commonness of more taxa than those for which it increased commonness. Second, despite the differences across taxa in responses of commonness to warming and herbivore exclusion, warming combined with herbivore exclusion elicited more changes in commonness than any other treatment. Third, commonness became increasingly right-skewed under herbivore exclusion, indicating increasing dominance by common taxa in the absence of herbivory, and less right-skewed under grazing, and these trends were evident under both warmed and ambient conditions. These three insights suggest that conservation of large herbivores may be crucial to maintaining the compositional integrity of arctic tundra communities under future warming through prevention or mediation of adverse effects on rare taxa. Finally, commonness of two of the most common taxa, the deciduous shrubs *B. nana* and *S. glauca*, increased under all but one treatment combination, and the rate of increase in their commonness scaled with their baseline commonness early in the experiment. Conversely, rates of decline in commonness of the rarest taxa scaled with their baseline rarity. This suggests that commonness itself may be an important predictor of trends in commonness under changes in both abiotic and biotic environmental conditions.

An important exception is the sitewide decline in commonness of graminoids, the second most common taxonomic group at the study site. Hence, while all rare taxa declined under at least one treatment combination in this experiment, graminoids in this case represent the decline of a common taxon under all experimental treatment combinations. While graminoids did not respond to warming itself, this group declined strongly under herbivore exclusion, which, we presume, is the ultimate driver of the declining commonness of this taxon at the study site. Caribou and muskoxen can slow or reverse shrub encroachment into graminoid lawns through both herbivory and trampling^29^, and the decline in commonness of graminoids at this study site may relate to the marked decline in abundance of caribou over the course of this experiment^36^.

In fact, we suggest that an interaction among several background conditions at the site has likely contributed to an increase in deciduous shrubs, and that this, in turn, has contributed to the decline in commonness of both graminoids and most of the rare plant taxa at the site. Specifically, local July mean temperature has increased by approximately 1 °C since the inception of this experiment^37^, caribou abundance has declined to approximately 150 individuals from a peak of approximately 600 individuals, and muskox abundance has increased from approximately 20 to 55 individuals at the site^36^. While background warming may have increased shrub abundance just as our experimental warming did (Fig. 1), opposing trends in herbivore abundances may have interacted in complex ways to further promote shrub increases, to the detriment of graminoids and rarer taxa. The overall decline in herbivore abundance at the site may have released deciduous shrubs from a browse trap^38^, allowing them to increase in abundance. And this effect is likely due mainly to a reduction in growing season browsing pressure by caribou, which tend to migrate into the study site in early spring and out of it in mid-summer^39^. Additionally, the increase in abundance of muskoxen at the site, which are resident at it year-round, has likely increased browsing pressure outside of the growing season, when apical stems, rather than leaves, are consumed. Such browsing action can promote stem bifurcation and canopy expansion the following growing season^40,41^. Finally, increases in deciduous shrub abundance can alter the local environment in disadvantageous ways for graminoids and forbs, including through shading, leaf litter deposition, and modification of the soil micro-environment and soil microbial activity^15,28,29,33,42,43^. Considering that numerous caribou and muskox populations are in decline across the Arctic^44–46^, we urge increasing focus on the manner in which commonness and rarity of tundra plants will be altered by interactions between climate change and variation in herbivore abundance.

## Methods

### Study site and experimental design

The study site, experimental design, and annual sampling protocol have been described in previous publications^15,22,47^ but a summary will be provided here. The experiment was conducted in a remote study site approximately 20 km northeast of Kangerlussuaq, Greenland, at 67.11° N latitude and 50.34° W longitude, approximately 160 km inland from Baffin Bay. Annual growing season (May through July) mean temperature and total precipitation at the study site during the duration of this experiment (2002–2017) were 8.62 ± 0.20 °C and 43 ± 6.78 mm, respectively^47^. The surrounding area has functioned as an important caribou (*Rangifer tarandus*) migration corridor, calving ground, and Indigenous Peoples hunting site for at least approximately 4000 years^48^, and was designated as a UNESCO World Heritage Site, Aasivissuit—Nipisat, by the United Nations in 2018. Caribou are present in greatest numbers seasonally, with most of the animals that use the site migrating into it during late winter and early spring and migrating out of it in mid to late summer; some male caribou remain at the site through winter. Muskoxen (*Ovibos moschatus*) are present at the site year-round. Arctic hares (*Lepus arcticus*) and rock ptarmigan (*Lagopus muta*) occupy the site in low numbers. In contrast to other locations in the Arctic where they are important herbivores, this site does not harbor voles or lemmings.

In June 2002 we erected six exclosures constructed of woven wire fencing material supported by steel t-posts; each exclosure was circular and measured 800 m^2^. Adjacent to each exclosure, and separated from it by approximately 20–50 m, we located a comparable control site. Exclosure sites and adjacent control sites covered a range of elevations from approximately 275–300 m above sea level. In early May 2003, prior to onset of the plant growing season, we installed passive, open-topped warming chambers constructed of UV neutral glazing material on three plots inside and three plots outside of one exclosure site and three plots inside and four plots outside of a second exclosure site. In early May 2004, we added three warming chambers inside and three warming chambers outside one of the sites equipped in 2003, and we installed an additional three warming chambers on plots inside and three warming chambers on plots outside of a third exclosure site, thus resulting in a total of 12 warmed plots distributed among three exclosure sites and 13 warmed plots distributed among three control (grazed) sites. An ambient (control) plot was located near, but not closer than 2 m to, each warmed plot, thus resulting in 25 warmed plots and 25 ambient plots distributed among three exclosures and adjacent grazed sites. No plot was located closer than 2 m to the edge of any exclosure. Warming chambers were constructed according to the International Tundra Experiment (ITEX) protocol^49^, were 1.5 m in basal diameter, and encompassed 1.77 m^2^. Warming chambers were installed in early May each year, anchored to plots using metal garden stakes, and removed annually at the time of vegetation sampling, which was intended to coincide with peak aboveground abundance at mid to late July in most years (except in 2006, when sampling was conducted in mid-June, and in 2003 and 2011 when sampling was conducted in mid-August)^47^. Warming chambers significantly elevated near surface temperature by approximately 1.5–3.0 °C, and resulted in a non-significant reduction of soil moisture^22,50^.

### Vegetation sampling

Vegetation sampling was conducted non-destructively using a square Plexiglas tabletop point frame on adjustable aluminum legs. The point frame measured 0.25 m^2^ and was centered within each plot for sampling. The corners of each plot were equipped with hollow aluminum tubes sunk into the soil surface at the cardinal directions, and the legs of the point frame were inserted into these tubes to ensure consistent orientation and location of the frame during sampling. Once the frame was positioned, a steel welding pin was lowered through each of 20 randomly located holes in the point frame tabletop, and each encounter by the tip of the pin with vegetation was recorded until the pin struck soil, litter, or rock. In 2003 and 2004, vegetation was recorded at the species level for deciduous shrubs (*Betula nana* and *Salix glauca*) and at the functional group level for graminoids (including grasses, rushes, and sedges of the genera *Calamagrostis* sp., *Poa* sp., *Festuca* sp., *Hierochloë* sp., *Trisetum spicatum*, *Luzula* sp., *Carex* sp., and *Kobresia* sp.), forbs, mosses, lichens, and fungi. Beginning in 2005, vegetation was recorded at the species level for forbs, in addition to deciduous shrubs, and at the genus level for lichens (*Peltigera* sp.), fungi [*Calvatia* sp.; most likely *C. cretacea*^51^], and mosses (*Aulacomnium* sp.). Graminoids were not resolved to the genus or species levels due to concerns about consistent identification. All taxa were identified in the field by the authors on the basis of visual inspection of live individuals in consultation with reference guides^52–55^. In adherence with the Guidelines for Professional Ethics established by the Botanical Society of America, sampling and identification were done non-destructively, and no voucher specimens were collected.

### Commonness estimation

Ecologically meaningful estimation of commonness is inherently relative; a taxon is only common or rare in relation to other taxa^5^. While there exist a considerable array of quantitative indices of commonness^56^, we opted for one that integrates abundance and occurrence by assigning equal weight to each. Using annual abundance sums obtained during point frame sampling, we calculated commonness for each taxon as the product of its proportional abundance across all plots within each treatment and its proportional occurrence across all plots within each treatment. Hence, the commonness (*C*) of an individual taxon, *i*, in a given year, *t*, can be expressed as the product of its proportional abundance (*A*) and proportional occurrence (*O*) in that year:

1 $$C_{it} = A_{it} *O_{it}$$

in which proportional abundance of taxon *i* in year *t* is the sum of point frame pin intercepts, *h*, for that taxon in that year across all plots sampled that year divided by the total number of point frame pin intercepts, *H*, of live vegetation biomass recorded across all plots sampled that year:

2 $$A_{it} = h_{it} /H_{t}$$

and in which proportional occurrence of taxon *i* in year *t* is the sum of the number of plots, *p*, on which point frame pin intercepts of taxon *i* were recorded in year *t* divided by the total number of plots, *P*, sampled in year *t*:

3 $$O_{it} = p_{it} /P_{t}$$

This index was used to estimate taxon-specific commonness within each experimental treatment combination (i.e., exclosed ambient, exclosed warmed, grazed ambient, and grazed warmed treatments), as well as across the entire site (sitewide commonness) for derivation of baseline commonness. To derive baseline commonness for subsequent analysis of its contribution to taxon-specific trends in commonness over the course of the experiment, we used sitewide commonness of each taxon in the year 2006. As described above, greater taxonomic resolution beyond functional group was not widely applied in our sampling until the third year of the experiment, 2005. However, we decided against using 2005 as a baseline for commonness at the site because it also happened to be the final year of a two-year outbreak of caterpillar larvae of a noctuid moth, *Eurois occulta*, that reduced aboveground abundance of nearly all taxa on our plots^22,57^. Except for the fungus *C. cretacea*, all taxa, whether recorded by pin intercepts during point-frame sampling or not, were observed on at least one plot under each of the four experimental treatment combinations. The rarest forb in this study, *Pyrola grandiflora*, was observed on a single plot under each of the exclosed ambient, exclosed warmed, and grazed warmed treatments, and on two plots under the grazed warmed treatment, but was not recorded during point frame sampling of exclosed ambient or grazed ambient plots. Hence, any conclusions about the effects of warming on this species must be limited. Similarly, the lichen *Peltigera* sp., which was also very rare in this study, was recorded during point frame sampling on plots under each treatment combination, but was not detected by sampling on exclosed warmed plots after 2005 even though it was observed on one exclosed warmed plot after that. This might be considered corroboration of the negative effect on this genus of warming under herbivore exclusion reported in the Results, but caution may also be warranted. The fungus *C. cretacea* first appeared under the grazed ambient treatment in 2008 and then under the exclosed ambient treatment in 2012, but was not recorded under the grazed warmed or exclosed warmed treatments. This might in and of itself suggest a negative effect of warming on the establishment or occurrence of this species, or fungi in general, and might be consistent with limiting effects of reduced moisture availability under warming. However, we urge caution with this interpretation because fungi may not form fruiting bodies every growing season, and such fruiting bodies may emerge aboveground in different locations from one growing season to the next, thereby potentially confounding repeated detection by sampling methods such as ours.

### Analysis of experimental treatment effects on plant functional group abundance

We used a Gaussian generalized linear model (GLM) with an identity link function to analyze variation in functional group abundance among experimental treatment combinations. This GLM included total annual abundance, for the period 2003–2017, of deciduous shrubs (comprising summed abundances of *Betula nana* and *Salix glauca* leaf and stem point frame pin intercepts), graminoids (comprising all grass, rush, and sedge tissue point frame pin intercepts), forbs, mosses, lichens, or fungi, in separate models with the two experimental treatments (warming and herbivore exclusion) and their interaction as factors, year as a factor, and day of year of sampling as a continuous covariate. Significance of individual treatment effects of warming and herbivore exclusion, as well as their interaction, was determined based on Wald Chi-square statistics and associated two-tailed *P*-values (with significance indicated at *P* ≤ 0.05).

### Analysis of experimental treatment effects on commonness

Analyses of commonness data were performed at higher taxonomic resolution than were analyses of abundance data, and so were limited to analysis of data from the last 12 years of the experiment, 2006–2017. Using Eq. (1), commonness was estimated for 14 taxa, including two species of deciduous shrubs, *Betula nana* and *Salix glauca*; graminoids, comprising at least eight non-distinguished genera of grasses, rushes, and sedges listed above in the sub-section Vegetation sampling; eight species of forbs, including *Equisetum arvense*, *Stellaria longipes*, *Cerastium alpinum*, *Bistorta vivipara*, *Draba nivalis*, *Campanula gieseckiana*, *Viola canina*, and *Pyrola grandiflora*; one genus of moss, *Aulacomnium* sp.; one genus of fungus, *Calvatia* sp.; and one genus of lichen, *Peltigera* sp.

We first investigated general characteristics of and treatment effects on commonness across the study site. We examined the skewness of commonness to determine whether the distribution of the 14 focal taxa was significantly right-skewed, indicating greater numbers of rare than of common taxa^2^. We obtained an estimate of skewness and its standard error across pooled data for the period 2003–2017, derived a 95% confidence interval, and compared it to zero. Next, we examined experimental treatment effects on sitewide commonness. To do this, we used a Gaussian GLM with identity link function to analyze pooled commonness of all taxa for the period 2006–2017, with commonness as the dependent variable and the two experimental treatments and their interaction as factors, year as a factor, taxon as a factor, and day of year of sampling as a covariate. We determined significance of individual treatment effects and their interaction by examining Wald Chi-square statistics, with significance indicated if the two-tailed *P* ≤ 0.05. We then tested for experimental treatment effects on individual taxa using the same analytical approach, but with taxon-specific commonness as the dependent variable, and treatment and year as factors, with day of year of sampling as a covariate.

### Analysis of trends in commonness and skewness of commonness over the last 12 years of the experiment

We next investigated whether common and rare taxa displayed different trends in commonness over the course of the last 12 years of the experiment. This was motivated by a presupposition that warming and/or herbivore exclusion might have differentially altered commonness of common vs. rare species. We first examined linear trends in sitewide commonness of all 14 taxa pooled across experimental treatments by testing for significance of linear regressions of taxon-specific commonness vs. year for the period 2006–2017. We then conducted the same analysis for each taxon individually under each experimental treatment combination to determine whether our experimental manipulations contributed to trends differentially in common vs. rare taxa. We then investigated whether the distribution of commonness across the 14 focal taxa displayed directional change over the course of the final 12 years of the experiment, and whether it might have done so differently in relation to experimental treatment combinations. To do this, we tested for significance of linear regressions of treatment-specific skewness of commonness vs. year for the period 2006–2017. Finally, we examined whether trends in commonness were related to baseline commonness for the 13 taxa resolved to the genus or species level, excluding graminoids because this group comprised multiple unresolved genera. This analysis was motivated by interest in determining whether taxa that were common at the beginning of the experiment tended to become more common and taxa that were rare at the beginning of the experiment tended to become rarer, thus indicating that degree of commonness itself might be an important driver of changes in commonness over the course of a multi-annual experiment such as ours. To do this, we fit a non-linear regression model using a von Bertalanffy equation to quantify the relationship between taxon-specific commonness trend (standardized coefficient from the regression of commonness vs. year, ranging between − 1 and 1) and baseline commonness by treatment. This equation took the form:

4 $$Y = 1 - \left( {1 - a} \right)e^{ - bX}$$

In which *Y* = taxon- and treatment-specific commonness trend, estimated in this case using the standardized coefficient from a linear regression of commonness of taxon *i* under a given experimental treatment combination vs. year; *a* = the Y-intercept; *b* = the slope; and *X* = baseline commonness of taxon *i* under the same treatment combination in 2006. Significance of regressions for each treatment was determined by calculating an *F*-statistic using corrected model sums of squares, error sums of squares, model degrees of freedom, and error degrees of freedom. Non-linear regression models were considered significant if the *F*-associated *P* ≤ 0.05.

## Supplementary Information

Supplementary Information.
