# Supplementary material for: Large herbivores facilitate the persistence of rare taxa under tundra warming
Source: Sci Rep. 2022 Jan 25;12:1292. doi: 10.1038/s41598-022-05388-4 (PMC8789846; doi:10.1038/s41598-022-05388-4)
Supplement: Supplementary file 1 — Supplementary Information. [file 41598_2022_5388_MOESM1_ESM.pdf]

Supplemental Table S1. Wald Chi-Square statistics for taxon-specific generalized linear models of commonness. Factors included experimental treatment and year, while day of year of sampling was included as a continuous covariate. See Methods for model details. Bold font and bold arrows indicate significance at $P \leq 0.05$.

	Experimental Treatment			Direction of treatment effect on commonness	
Taxon	Warming	Exlcosure	Warming * Exclosure	Warming	Exclosure
Betula nana	**183.8**	**658.9**	**141.4**	**↓**	**↑**
Salix glauca	**28.98**	**203.2**	**47.4**	**↑**	**↓**
Graminoids	**9.23**	**332.5**	0.986	**↓**	**↓**
Equisetum arvense	0.23	**10.69**	0.15	↓	**↓**
Aulacomnium sp.	0.54	**14.8**	**72.6**	↓	**↓**
Cerastium alpinum	**4.1**	**9.24**	0	**↑**	**↓**
Stellaria longipes	1.18	0.04	**4.75**	↑	↓
Bistorta vivipara	**13.3**	**7.88**	0.49	**↓**	**↓**
Draba nivalis	**8.74**	**20.8**	1.85	**↑**	**↓**
Viola canina	0.51	0.002	0.009	↓	↑
Campanula gieseckiana	1.68	**3.75**	0.94	↓	**↑**
Peltigera sp.	0.09	0.17	**6.37**	↓	↓
Pyrola grandiflora	**8.86**	3.64	3.64	**↑**	↑
Calvatia cretacea	**7.32**	0.02	0.02	**↓**	↑
